# American Academy of Dental Science

**Published:** 1871-11

**Authors:** 


*American Academy of Dental Science.*—From the "Boston Post," we take the following account of the fourth anniversary dinner of this Society, served at the Parker House:

"Dr. Daniel Harwood presided. Prayer was offered by the Rev. Theodore W. Snow. After doing full justice to the bill of fare, the first speech was made by Dr. A. P. Stevens, of Portsmouth, N. H., who spoke of the immense amount of incompetency among many claiming to be dentists throughout the land, and of the necessity of thorough dental education. Rev. Mr. Snow said that he could revert to dental operations performed by Dr. Randall, in Boston, fifty-five years ago, who told his patients, to relieve their fears, that his instruments were wrapped in silk. He knew of no more honorable occupation than a competent dentist, and he felt a sincere regard for the highest interests of the Society of Dentistry. Dr. E. T. Wilson spoke of the American Academy of Dental Science as an institution to be honored throughout the country. Prof. J. H. McQuillen, of Philadelphia, acknowledged in a happy manner the kindness with which he had been received in Boston. Drs. M. B. Mead and F. N. Seabury, of Providence, responded for the profession in Rhode Island. Dr. James McManus, President of the Connecticut State Dental Society, rejoiced in the privilege of being present to participate in the exercises of the anniversary. Dr. Harwood said that he began forty-two years ago the practice of dentistry in Boston, and had always labored for the elevation of the dental profession. While conservative, he believed in improvement and progress. Dr. J. H. Foster, of New York, urged a compulsory system of education as a remedy for the existing evils. Dr. C. E. Francis, President of the Odontological Society, of New York, spoke of dental societies as the very backbone of the profession. It is at such gatherings that the members bring their best thoughts and keep up the spirit inculcated at the colleges. He thought we could not be too particular in the selection of dental students. Dr. Joshua Tucker made a few remarks and was followed by Dr. E. G. Tucker, who said it always gave him pleasure to recur to the old times of Harwood and the elder Tucker, and to trace the branches from those pioneers to the present time. The great and growing fact on which the progress of dental science depends is now conceded by educated men to be the union of theoretrical and practical knowledge, and it remains to be seen how long the unthinking masses will prefer quackery to scientific treatment. Dr. L. D. Shepard, of Boston, spoke of the kindly feeling existing in the Academy between the old and the young practitioners.

"Dr. E. N. Harris, the Secretary, read letters expressing their interest, but regretting their inability to be present, from Drs. W. W. Allport, of Chicago; H. M. Bowker, of Montreal; Samuel S. White, of Philadelphia, and O. W. Holmes and Winslow Lewis, of Boston. Mr. Charles H. Frothingham, being present as an invited guest, said that he did not think that dentists as a body were unpatriotic, but he was surprised that no one had proposed the customary toast to the President of the United States, and in order they might not suffer in the minds of any by reason of this omission, he gave them, and thought there was especial appropriateness in so doing—"The health of the present Presi-Dent of the United States, Gen. Ulysses S. Grant, who from his numerous *Dent*-al connections and his great admiration for the family—as evinced by his marrying a Dent and appointing so many Dents to office—may be fairly considered the most distinguished Dentist in the land." The health of Gen Grant, "the great dentist was then drunk amid much applause.

"Other speeches were made by Drs. H. F. Bishop, of Worcester; John Clough, of Woburn, and J. L. Williams, W. W. Codman, A. T. Emery, W. D. Tucker and C. P. Wilson, of Boston.

